# Phosphatidylserine on viable sperm and phagocytic machinery in oocytes regulate mammalian fertilization

**DOI:** 10.1038/s41467-019-12406-z

**Published:** 2019-10-01

**Authors:** Claudia M. Rival, Wenhao Xu, Laura S. Shankman, Sho Morioka, Sanja Arandjelovic, Chang Sup Lee, Karen M. Wheeler, Ryan P. Smith, Lisa B. Haney, Brant E. Isakson, Scott Purcell, Jeffrey J. Lysiak, Kodi S. Ravichandran

**Affiliations:** 1 The Center for Cell Clearance, School of Medicine, University of Virginia, 1340 Jefferson Park Avenue, Pinn Hall, Charlottesville, VA 22903 USA; 2 Department of Microbiology, Immunology, and Cancer Biology, School of Medicine, University of Virginia, 1340 Jefferson Park Avenue, Pinn Hall, Charlottesville, VA 22903 USA; 3 Department of Urology, School of Medicine, University of Virginia, 1340 Jefferson Park Avenue, Pinn Hall, Charlottesville, VA 22903 USA; 4 College of Pharmacy and Research Institute of Pharmaceutical Sciences, Gyeongsang National University, 501 Jinju-daero, Jinju, Gyeongnam 52828 Republic of Korea; 5 Department of Molecular Physiology and Biological Physics, School of Medicine, University of Virginia, 1340 Jefferson Park Avenue, Pinn Hall, Charlottesville, VA 22903 USA; 6 Reproductive Medicine and Surgery Center of Virginia, 595 Martha Jefferson Dr., Charlottesville, VA 22911 USA; 7 Department of Biomedical Molecular Biology, Ghent University, and the UGent-VIB Center for Inflammation Research, Technologiepark 71, 9052 Ghent, Belgium

**Keywords:** Cell biology, Developmental biology, Germline development

## Abstract

Fertilization is essential for species survival. Although Izumo1 and Juno are critical for initial interaction between gametes, additional molecules necessary for sperm:egg fusion on both the sperm and the oocyte remain to be defined. Here, we show that phosphatidylserine (PtdSer) is exposed on the head region of viable and motile sperm, with PtdSer exposure progressively increasing during sperm transit through the epididymis. Functionally, masking phosphatidylserine on sperm via three different approaches inhibits fertilization. On the oocyte, phosphatidylserine recognition receptors BAI1, CD36, Tim-4, and Mer-TK contribute to fertilization. Further, oocytes lacking the cytoplasmic ELMO1, or functional disruption of RAC1 (both of which signal downstream of BAI1/BAI3), also affect sperm entry into oocytes. Intriguingly, mammalian sperm could fuse with skeletal myoblasts, requiring PtdSer on sperm and BAI1/3, ELMO2, RAC1 in myoblasts. Collectively, these data identify phosphatidylserine on viable sperm and PtdSer recognition receptors on oocytes as key players in sperm:egg fusion.

## Abstract (web summary)

Izumo and Juno are receptors on sperm and eggs respectively required for fusion, but other factors for sperm-egg fusion are poorly studied. Here, the authors report that phosphatidylserine, found mainly on cells marked for death, is also present on motile sperm, recognized by egg receptors, and is required for sperm-egg fusion.

## Introduction

Sexual reproduction requires a productive fusion between the haploid male and female gametes^1,2^. Prior to the fusion of the gametes, a critical step is the proper recognition between specific ligand(s) on the sperm and appropriate binding partner(s) on the egg. Recent studies both at the functional and structural levels have unequivocally established a critical role for the sperm surface protein Izumo1 and the corresponding GPI-anchored receptor Juno on the oocyte, with blocking or loss of either protein affecting fertilization^3,4^. Signaling downstream of Juno in oocytes is yet to be defined, as Juno is a GPI-anchored protein. Further, 3D structure studies^5,6^ suggest that the Izumo1:Juno interaction is unlikely to lead to fusion and, when Izumo1 was exogenously expressed on Cos-7 cells^7^, oocyte binding to these cells occurred but did not proceed to fusion. The tetraspanin family member CD9 on the oocyte has also been linked to mammalian fertilization^8^. CD9 has no known ligand and it is thought to modify the membrane curvature^9,10^. Thus, it has been suggested that other players on both sperm and the oocyte likely contribute toward gamete fusion (after the Izumo1:Juno interaction)^1,2^.

Here, using complimentary approaches on the oocyte and sperm, we show that Phosphatidylserine (PtdSer), exposed on viable sperm, is recognized by specific receptors located on the microvilli of the oocyte to promote sperm:egg fusion. The signaling pathway ELMO1/RAC1, downstream of the PtdSer receptors BAI1/3, also participates in this event. This pathway is also conserved in the fusion of sperm with myoblasts. Taken together, our results shed light into the molecular mechanism of sperm:egg fusion.

## Results

### PtdSer exposed on viable sperm is required for fertilization

**Fig. 1:**
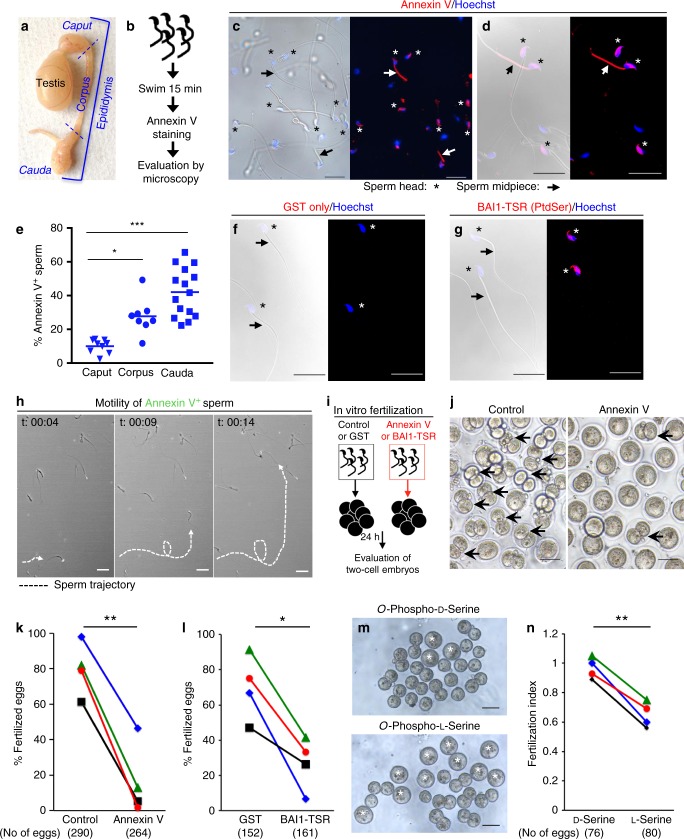
Phosphatidylserine on live sperm is important for in vitro fertilization. **a** Depiction of the mouse testis and epididymis. **b** Sperm from different regions of the epididymis were allowed to swim/disperse in TYH + BSA medium, stained with Annexin V and Hoechst, and evaluated by microscopy. **c**, **d** Annexin V staining of sperm. Asterisks denote sperm heads, and arrows midpiece. Scale bar, 20 μm. **e** Percentage of Annexin V + sperm from the caput (*n* = 9 mice), corpus (*n* = 8 mice), and cauda (*n* = 15 mice) epididymis, with each dot representing one mouse (six independent experiments). *p < 0.05, ***p < 0.001 (one-way non parametric ANOVA was followed by Kruskal–Wallis test for multiple comparisons). **f**, **g**, Sperm from cauda epididymis were incubated with 50 μg/ml GST only **f** or GST-BAI1-TSR **g**, washed, fixed and visualized by GST immunofluorescence. Scale bar, 20 μm. **h** Snap shots of a movie depicting motility of live Annexin V + (green) sperm (t: time in min). The trajectory of a single sperm is traced by a white dotted line. Scale bar, 30 μm. **i** Schematic of the in vitro fertilization assay: cumulus oocyte complexes isolated from wt super-ovulated females were incubated with caudal sperm previously capacitated, in the presence or absence of 10 μg/ml Annexin V, 50 μg/ml GST, or 50 μg/ml GST-BAI1-TSR. The percentage of fertilized eggs (two-cell embryos) was evaluated after 24 h. **j** Multiple two-cell embryos fertilization with control sperm (left panel, arrows), whereas fewer fertilized eggs were observed with Annexin V (right panel, arrows). Scale bar, 100 μm. **k**, **l** Annexin V masking of PtdSer on sperm (**k**, *n* = 4 experiments) or GST-BAI1-TSR (**l**, *n* = 4 experiments) reduces fertilization. The total number of eggs analyzed is shown in parentheses. Each line represents one experiment and the matching experiments are shown (shape and color). Error bars are s.e.m. *p < 0.05, **p < 0.01 (Two-tailed unpaired Student’s *t* test). **m**, **n** Greater unfertilized oocytes (asterisks) seen after competition with *O*-Phospho-l-Serine compared with *O*-Phospho-d-Serine (top). Scale bar, 100 μm. Each line represents one experiment and matching experiments are shown with the same shape and color (*n* = 4 experiments). Error bars are s.e.m. **p < 0.01 (Two-tailed unpaired Student’s *t* test). Source Data are provided in the Source Data File

As part of our studies on apoptotic germ cell clearance in the testes^11^, we noticed Annexin V staining on freshly isolated sperm from the cauda epididymis (Fig. 1a, b), suggestive of PtdSer exposure (Fig. 1c, d). Although PtdSer has been noted on sperm previously^12–14^, it was considered to mark dead or non-viable sperm because PtdSer is a key eat-me signal on cells undergoing apoptosis, which facilitates recognition and uptake by phagocytes^15^. Interestingly, PtdSer can also be transiently exposed on viable cells in certain conditions (such as activated B and T cells, myoblasts, or macrophages^16–19^), and therefore, we further investigated the relevance of PtdSer exposure on the sperm.

Annexin V staining was prominently seen both on the sperm head and the midpiece, but was absent in the tail (Fig. 1c, d). During spermatogenesis, after exiting the testis, sperm transits through different segments of the epididymis: the caput, the corpus and the cauda (Fig. 1a). Classical experiments have shown that only the caudal sperm is capable of fertilization^20^. Therefore, we assessed PtdSer exposure on sperm as it transits through the epididymis. We noticed a progressive increase in PtdSer exposure on sperm isolated from different segments of the epididymis, with the cauda epididymis, which contains the fertilization-competent sperm, displaying the highest percentage of PtdSer-positive sperm (Fig. 1e). This also indicates that PtdSer externalization is not merely an effect of sperm isolation. When we addressed whether PtdSer exposure on sperm changes with capacitation, a process known to occur in the female reproductive tract^21^, we found a further increase in the percentage of PtdSer-positive sperm after capacitation in vitro (Supplementary Fig. 1a*)*. The acrosome reaction is another process that occurs on sperm in the female tract. When we induced the acrosome reaction in vitro with the ionophore A23187, PtdSer continued to be detectable on sperm. Further, as Izumo1 is exposed on caudal sperm after the acrosome reaction and is a central player in fertilization^4^, we asked whether PtdSer colocalizes with Izumo1 on sperm. Izumo1 (detected via antibody), as well as PtdSer (via Annexin V), were colocalized in the equatorial region of the sperm head (known to be involved in sperm:oocyte fusion^2^) (Supplementary Fig. 2). As Annexin V can bind both PtdSer and phosphatidylethanolamine (PtdEtn)^22^, we tested PtdSer exposure on the sperm using a second reagent. We have previously established that the soluble extracellular fragment of the PtdSer recognition receptor BAI1 (BAI1-TSR fused to the glutathione-*S*-transferase; GST) binds PtdSer but not PtdEtn^23^; BAI1-TSR (but not the control GST protein) preferentially bound to the sperm head without significant midpiece binding (Fig. 1f, g). Consistent with this observation, when we used Duramycin, which binds PtdEtn but not PtdSer^22^, the binding was noted prominently in the midpiece with much less binding to the head (Supplementary Fig. 1b). These data suggested specific exposure of PtdSer on the heads of freshly isolated sperm from the cauda epididymis.

To address whether the sperm with PtdSer exposed on their surface are viable, we performed time-lapse microscopy, revealing that the PtdSer^+^ sperm were motile (Fig. 1h, and Supplemental movie 1). Further, when we analyzed the expression on the caudal sperm of cleaved caspase 3 (CC3), a marker for apoptosis, the Annexin V^+^ sperm were negative for CC3 (Supplementary Fig. 1c). Only after 24 h incubation ex vivo, some of the sperm showed CC3 staining (Supplementary Fig. 1d). These data suggested that PtdSer is exposed on the surface of viable and motile sperm.

We next asked whether the PtdSer exposure on sperm is functionally important for fertilization. To test this, we performed in vitro fertilization using capacitated caudal sperm and oocytes from super-ovulated C57BL/6 female mice, and quantified the emergence of two-cell embryos (see schematic in Fig. 1i). We deliberately chose three different reagents to target PtdSer, as each have unique features that are complementary, and can inform us better about the role of PtdSer during fertilization: (1) Annexin V (which has the highest affinity); (2) BAI1-TSR peptide derived from the extracellular region of the PtdSer receptor BAI1 fused to the GST (lower affinity than Annexin V but greater specificity for PtdSer); and (3) the soluble head group of the lipid phosphatidylserine, which has the lowest affinity of the three blocking agents but can act as a competitive inhibitor for PtdSer recognition receptors. Pleasingly, all three reagents supported the hypothesis that PtdSer on sperm contributes to fertilization. Annexin V caused > 85% reduction in fertilization in three out of four independent experiments (Fig. 1j, k). Masking PtdSer on sperm with BAI1-TSR also significantly reduced fertilization, and consistent with the BAI1-TSR being of lower affinity than annexin V, the inhibition of fertilization with BAI1-TSR was less pronounced (Fig. 1l). Addition of the soluble Phospho-l-Serine head group also resulted in significant reduction in fertilization in every experiment (Fig. 1m, n). The partial inhibition (30–40%) was expected as the monomeric soluble head groups of PtdSer have to compete against multi-valent PtdSer recognition on the sperm surface. The advantage of using the Phospho-l-serine blocking is that we could directly compare its effect against the stereoisomer Phospho-d-Serine as a control (carrying the same charge), and this did not inhibit fertilization. Of note, we confirmed that progressive motility of the sperm was not affected after masking PtdSer with Annexin V or BAI1-TSR (Supplementary Fig. 1e, f). Collectively, these three approaches suggest that recognition of PtdSer on sperm (in addition to the well-described Izumo1) can contribute to in vitro fertilization.

### PtdSer receptors on oocytes contribute to fertilization

**Fig. 2:**
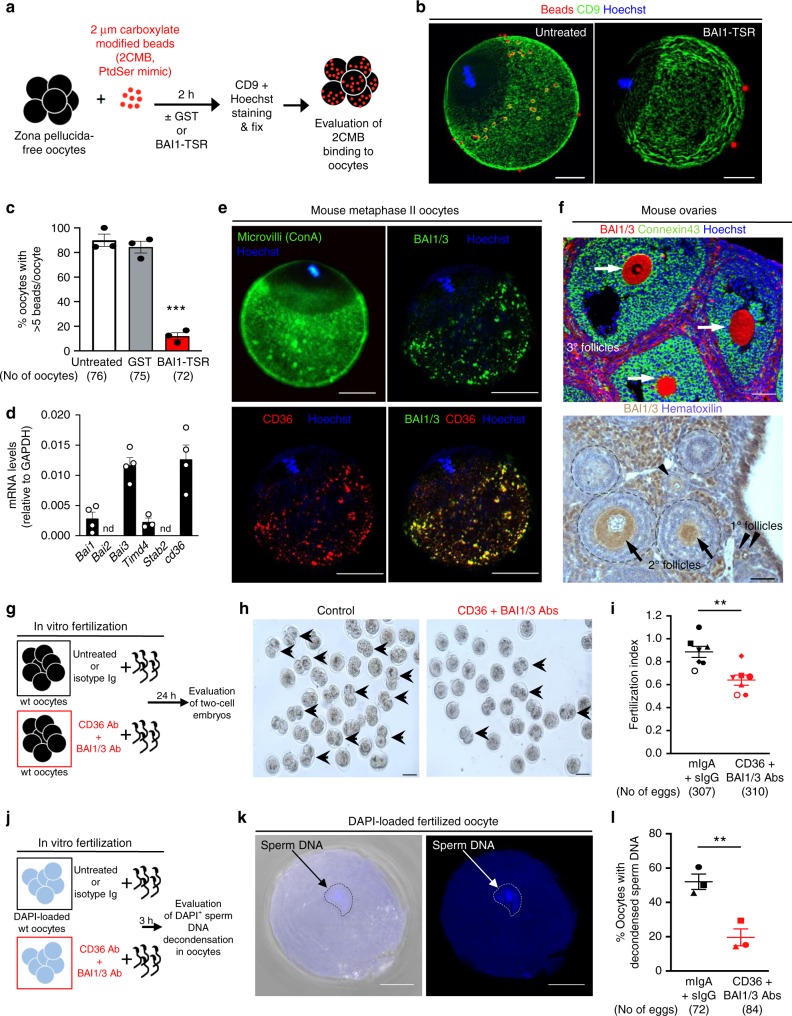
BAI1/3 and CD36 are expressed on oocytes and contribute to fertilization. **a** Schematic for the assay to determine PtdSer recognition moieties on mouse oocytes. **b** Oocytes-bound multiple untreated carboxylate beads within the microvillar region (left), but not the BAI1-TSR-treated beads (right). Scale bar, 20 μm. **c** Summary of three experiments using carboxylate binding oocytes. ***p < 0.001 (Two-tailed unpaired Student’s *t*-test). **d**, PtdSer expression (qPCR) on cumulus-free Metaphase II oocytes from wild-type female mice (*n* = 4 experiments). **e** Concanavalin A (ConA) staining (marking microvilli) overlap with staining for BAI1/3 and CD36 on ZP-free oocytes. Scale bar, 20 μm. **f** Immunofluorescence (top) and immunohistochemistry (bottom) staining of mouse ovaries with BAI1/3 antibodies. Connexin-43 (green) marks follicular cells, ovarian follicles (dotted lines) are indicated. Arrows: oocytes in secondary or tertiary follicles; arrowheads: oocytes in primary follicles. Scale bar, 50 μm. **g**, Schematic of fertilization assays assessing the role of BAI1/3 and CD36. **h**, **i** Multiple two-cell embryos are observed in the control group (left, arrows) but reduced after CD36 and BAI1/3 antibody treatment (right). Scale bar, 100 μm. The compilation of data from 7 independent experiments is shown in **i**. Each dot represents one experiment, with matching experiments shown (shape and color). sIgG, sheep IgG, mIgA, mouse IgA. **p < 0.05 (two-tailed unpaired Student’s *t* test). **j** Schematic of in vitro fertilization assays to test BAI1/3 and CD36 in sperm entry. Untreated or antibody-treated oocytes were loaded with DAPI, and mixed with sperm. The percentage of oocytes with decondensed sperm DNA was evaluated after 3 h. **k**, **l** A representative image of a fertilized oocyte with one decondensed sperm DNA **k**. Scale bar: 20 μm. Quantitation of oocytes with decondensed sperm DNA after blocking with CD36 and BAI1/3 antibodies **l**. Each dot represents one experiment (*n* = 3 independent experiments) and the matching experiments are shown (shape and color). **p < 0.01 (two-tailed unpaired Student *t* test). Data are presented as mean ± s.e.m. Source Data are provided in the Source Data File

To test whether the surface of oocytes contains potential PtdSer-binding sites, we took a simplified approach using the binding of the fluorescently labeled 2 µm carboxylate modified beads (2CMB), which are known to bind Annexin V and compete with PtdSer-exposing apoptotic cells^23^. Zona pellucida (ZP)-free oocytes were isolated from wild-type C57BL/6 female mice and were incubated with red-fluorescent 2CMB for 2 h. At the end of the incubation period, the oocytes were stained with CD9 antibody to identify the microvillar region that is known to interact with sperm, fixed, and analyzed by microscopy (Fig. 2a). Oocytes readily bound multiple beads, and this interaction was restricted to the microvillus region of the oocytes (Fig. 2b). Importantly, this binding was significantly decreased when the beads were pretreated with the PtdSer-masking agent BAI1-TSR (Fig. 2b, c). Although PtdSer is an eat-me signal on apoptotic cells, extensive confocal sectioning of the bead-bound oocytes did not reveal obvious internalization of the beads under these conditions. These data indicated the existence of potential PtdSer-binding molecules in the microvillar region on the surface of oocytes. As masking PtdSer on sperm significantly reduced the fertilization rate, we hypothesized that the PtdSer recognition receptors on the oocytes may contribute to the steps toward sperm:egg fusion.

Several PtdSer recognition receptors with redundant functions have been identified on phagocytes to engage the PtdSer exposed on the apoptotic targets^23–26^. Therefore, we hypothesized that one or more such PtdSer recognition receptor(s) on the oocytes may engage the sperm during fertilization. In a previous bioinformatics analysis of oocyte genes, members of the BAI family as well as CD36 were reported to be expressed in both mouse and human oocytes^27^. When we assessed the mRNA expression of BAI family members and CD36, we found readily detectable expression of BAI1, BAI3, and CD36 in mouse oocytes (Fig. 2d). BAI members belong to the type II adhesion family of GPCRs (hence, also referred to as ADGBR family) with long extracellular region containing domains capable of directly binding PtdSer^23,25,28–32^; CD36 is a member of the scavenger receptor family, and has also been linked to the binding of PtdSer^24,33–35^. CD36 is also reported to function cooperatively with BAI1 on endothelial cells^36^. Immunofluorescence microscopy using antibodies, which recognize both BAI1 and BAI3 (referred to from here onwards as BAI1/3) or CD36, gave a prominent signal in the sperm-binding microvillar region (Fig. 2e); this staining pattern was also similar to the staining previously noted with concanavalin A^37^ (Fig. 2e), Juno and CD9^7,9^ (Supplementary Fig. 3a). When we assessed the expression of other known direct PtdSer-binding receptors, we found detectable expression of the message for *Timd4* but not *Stab2* (Fig. 2d). Among the TAM family of receptors that can also recognize PtdSer (indirectly, via the bridging molecules Gas6 or protein S^26,38^), *Mertk*, but not *Tyro3* and *Axl* were noted on oocytes^39^. Immunohistochemistry of whole mouse ovaries revealed that BAI1 expression is detectable in oocytes from the earliest stages of folliculogenesis, with positive staining from primordial follicles through tertiary follicles (Fig. 2f). Similarly, we could readily detect staining for BAI1/3 and CD36 on human oocytes (discarded/unused oocytes acquired from clinical in vitro fertilization procedures) (Supplementary Fig. 3b). Furthermore, expression of BAI1/3 on oocytes was detectable via immunohistochemistry on tissue sections of human ovary (Supplementary Fig. 3c).

In the efferocytosis field, the PtdSer recognition by PtdSer receptors is known to include redundant mechanisms, as the charged head group of the lipid PtdSer can be recognized in a polyvalent fashion by multiple receptors to provide sufficient avidity and specificity^38,40,41^. Therefore, we decided to test all of the five potential PtdSer receptors detected in oocytes—CD36, BAI1, BAI3, Tim-4, and Mer-TK—via approaches that target them either singly or in combination. Interestingly, CD36 has been shown to cooperatively function with BAI1 in endothelial cells^36^. Therefore, to test the potential multi-pronged interaction involving both CD36 and BAI1/3, we tested the effect of antibodies targeting CD36 or BAI1/3 (via antibody that recognizes both BAI1/BAI3), either alone or combination (see schematic in Fig. 2g). Although antibodies to either BAI1/3 or CD36 alone did not inhibit fertilization (Supplementary Fig. 3d), a combination of antibodies targeting both BAI1/3 and CD36 caused a reproducible and statistically significant inhibition of fertilization in vitro (Fig. 2h, i). As a positive control, antibody to Juno could strongly inhibit fertilization (Supplementary Fig. 3f). Next, we used a more direct assay for the sperm entry into oocytes. During fertilization, the nucleus of the sperm decondenses after entry into the oocyte cytoplasm^42–44^. This early step of fertilization can be scored using oocytes loaded with the DNA binding dye 4′,6-diamidino-2-phenylindole (DAPI), and the appearance of DAPI-stained decondensed sperm DNA (Fig. 2j)^42–44^. After establishing and validating this assay, we tested the efficacy of the BAI1/3- and CD36-blocking antibodies on the sperm entry into the oocyte. We found a 67% decrease in the percentage of oocytes with decondensed sperm DNA (Fig. 2k, l), suggesting that blocking three of the five PtdSer receptors (BAI1/BAI3, and CD36) expressed on oocytes can also impair fertilization in vitro, complementary to the masking of PtdSer on the sperm.

**Fig. 3:**
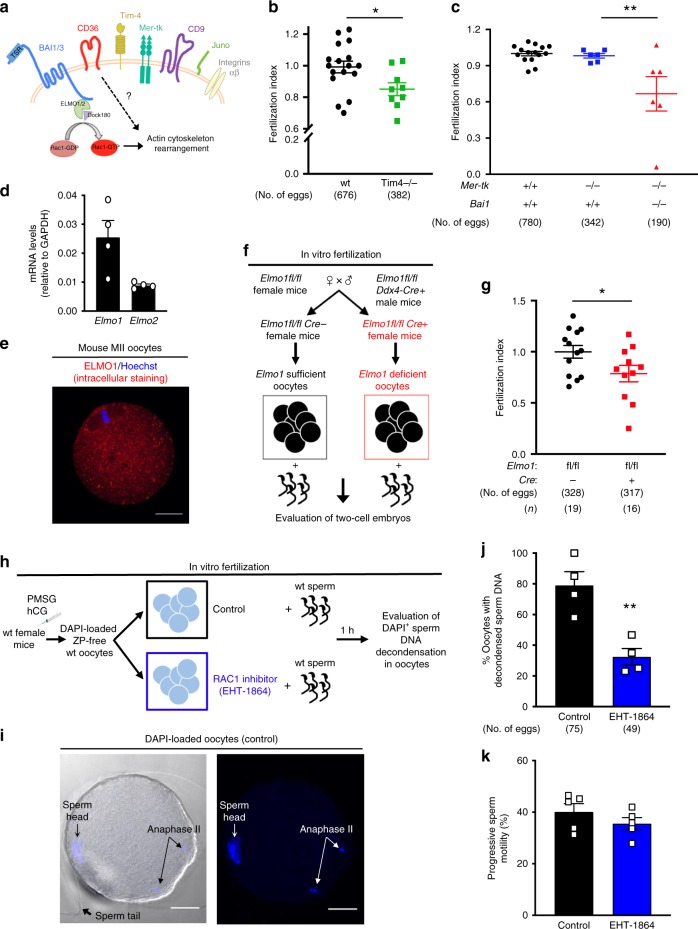
Genetic testing of PtdSer receptors and cytoplasmic signaling in oocytes. **a** Schematic of PtdSer receptors BAI1/3, the downstream ELMO-DOCK-RAC1 signaling pathway, and other receptors on oocytes. **b**, **c** The PtdSer receptors Tim-4, BAI1 and Mer-TK participate in fertilization. ZP-intact oocytes from wt or Tim-4^*−/−*^ mice **b** *Mer-tk* + / + Bai1 + / + , *Mer-tk*^*−/−*^ *Bai1* + / + , or *Mer-tk*^*−/−*^ *Bai1* + /- or *Mer-tk*^*−/−*^ *Bai1*^*−/−*^ **c** were mixed with wt sperm, and two-cell embryos evaluated at 24 h. Fertilization index is the percentage of fertilized eggs from the experimental group divided by percentage of fertilized eggs from the control group (wt mice). Each dot represents one mouse (**b**, *n* = 6 experiments including 17 wt mice and 9 Tim-4^*−/−*^ mice, **c**: *n* = 5 experiments including 15 wt mice, 6 *Mer-tk*^*−/−*^ *Bai1* + / + and 6 *Mer-tk*^*−/−*^ *Bai1*^*−/−*^), total number of eggs (parentheses). *p < 0.05 (**b**, two-tailed unpaired Student *t* test), **p < 0.01 (**c** one-way ANOVA followed by Dunnet’s multiple comparisons test). **d** *Elmo1* and *Elmo2* mRNA on cumulus-free Metaphase II oocytes. **e** Intracellular ELMO1 in isolated Metaphase II (MII) ZP-free oocytes. Scale bar, 20 μm. **f** Schematic for generation of oocyte-specific *Elmo1*-deficient mice. **g** Percentage of fertilized eggs after incubation of control (*Elmo1*^*fl/fl*^) or *Elmo1*-deficient (*Ddx4-Cre/Elmo1*^*fl/fl*^) oocytes with wt sperm (*n* = 6 independent experiments including 15 *Elmo1*^*fl/fl*^ mice and 12 *Ddx4-Cre/Elmo1*^*fl/fl*^ mice). Each dot represents 1 or 2 pooled mice. *p < 0.05 (Two-tailed unpaired Student’s *t* test). **h** Schematic for the evaluation early sperm entry into oocytes via DAPI staining of decondensed sperm DNA. ZP-free wt oocytes were incubated with RAC1 inhibitor (EHT-1864, 80 μm) and loaded with DAPI. After several washes, sperm were added, and the presence of internalized sperm with decondensed nuclei was evaluated after 1 h. **i** Control oocytes displaying the decondensation of the sperm DNA incorporated into the oocyte (arrow), whereas the sperm tail has not yet been internalized. DAPI also highlights the oocyte chromosomes in anaphase II indicating the resumption of meiosis II. Scale bar, 20 μm. **j**, Decreased percentage of oocytes with decondensed sperm DNA after RAC1 inhibition (*n* = 4 independent experiments). Numbers in parentheses reflect total number of eggs scored. **p < 0.01 (Two-tailed unpaired Student’s *t* test). **k** Sperm motility was not affected by RAC1 inhibition *p* > 0.05 (two-tailed unpaired Student’s *t* test). Data are presented as mean ± s.e.m. Source Data are provided in the Source Data File

Next, we wanted to genetically test the contribution of oocyte PtdSer receptors to fertilization. Because of the extensive functional redundancy among PtdSer recognition receptors it is widely reported that single knockout of PtdSer receptors often show partial defects in apoptotic cell clearance, and defects are better revealed by deletion of more than one receptor^45–48^. We tested three of the PtdSer receptors expressed on oocytes using single or double knockout mice: Tim-4, BAI1, and Mer-TK. Tim-4 directly binds PtdSer while Mer-TK binds PtdSer indirectly through the bridging molecules Gas6 or Protein S [note: oocytes also express Gas6^39^]. Mice deficient in Tim-4 alone showed a modest but statistically significant reduction in the percentage of fertilized eggs (Fig. 3b). We then tested the role of *Mertk* and *Bai1* genetically. We performed in vitro fertilization assays with oocytes isolated either from *Mertk*^*−/−*^ or *Bai1*^*−/−*^ mice, or mice double deficient for both *Mertk* and *Bai1*. Although oocytes isolated from single deficient mice (either *Mertk*^*−/−*^ or *Bai1*^*−/−*^) could be fertilized similar to wild-type eggs (Fig. 3c and Supplementary Fig. 3e), the double deficient *Mertk*^*−/−*^*Bai1*^*−/−*^ oocytes show a significant reduction in fertilization (Fig. 3c). Collectively, these data suggest that even with the considerable redundancy among the PtdSer receptor family, a statistically significant effect can be observed in in vitro fertilization with oocytes deficient in specific PtdSer recognition receptors.

### The BAI1/3-ELMO1-Rac1 signaling axis affects fertilization

With respect to signaling downstream of PtdSer recognition receptors, CD36 has a rather short cytoplasmic tail without an obvious direct signaling, and CD36 can cooperatively signal with BAI1^36^. Among the PtdSer receptors, signaling downstream of the BAI family members is one of the best characterized^23,25,49^. Both BAI1 and BAI3 have long cytoplasmic tails that associate with the adapter proteins ELMO1 and/or ELMO2 (depending on the cell type), with subsequent signaling (in complex Dock family proteins) and activation of the small GTPase RAC1^23,49–52^. GTP-bound active RAC1 promotes actin cytoskeletal remodeling during adhesion, phagocytosis, and cell:cell fusion events (Fig. 3a). In oocytes, we detected both *Elmo1* and *Elmo2* mRNA, with *Elmo1* expression higher than *Elmo2* (Fig. 3d). At the protein level, ELMO1 expression was readily detected by immunofluorescence in isolated oocytes (Fig. 3e). To assess the importance of ELMO1 in fertilization, we crossed mice carrying floxed *Elmo1* alleles (Elmo1^fl/fl^) with Ddx4-Cre mice^53^, which express the Cre recombinase specifically in oocytes from the earliest stages (Fig. 3f). We super-ovulated the *Ddx4-Cre*/*Elmo1*^*fl/fl*^ female mice, isolated the oocytes, and performed in vitro fertilization assays using caudal sperm from wild-type mice. We noted a significant reduction in in vitro fertilization with oocytes from *Ddx4-Cre/Elmo1*^*fl/fl*^ mice, compared with control mice (Fig. 3g). The partial reduction is consistent with the continued expression of ELMO2, which can substitute for ELMO1^11^. Incidentally, female nematodes lacking the ELMO homolog *ced-12* have been shown to have lower fecundity, with fewer progeny produced^51^.

ELMO proteins (together with Dock family members) function as upstream activators of the small GTPase RAC1, which regulates actin cytoskeletal rearrangements^51^ (Fig. 3a). As genetically testing the requirement for RAC1 is not feasible owing to the role of RAC1 during oocyte development and other steps after the sperm entry^54^, we took a pharmacological approach and used the sperm DNA decondensation assay to more directly score the sperm entry into oocytes. We treated oocytes with the RAC1 inhibitor EHT-1864 (see methods) and also added EHT-1864 during co-incubation of sperm and oocytes (note that both sperm and oocytes were harvested from wild-type mice) (Fig. 3h, i). EHT-1864 caused a significant reduction in the number of oocytes with decondensed sperm DNA (Fig. 3j). Importantly, this effect did not appear to be owing to the RAC1 inhibitor affecting sperm, as sperm incubated with EHT-1864 alone under the assay conditions showed no reduction in the motility (Fig. 3k). These data suggest that oocyte BAI1/3 and CD36, as well as the ELMO–RAC1 signaling module downstream of BAI1/3 contribute to the functional steps of fertilization.

### PtdSer-dependent fusion of sperm with skeletal myoblasts

**Fig. 4:**
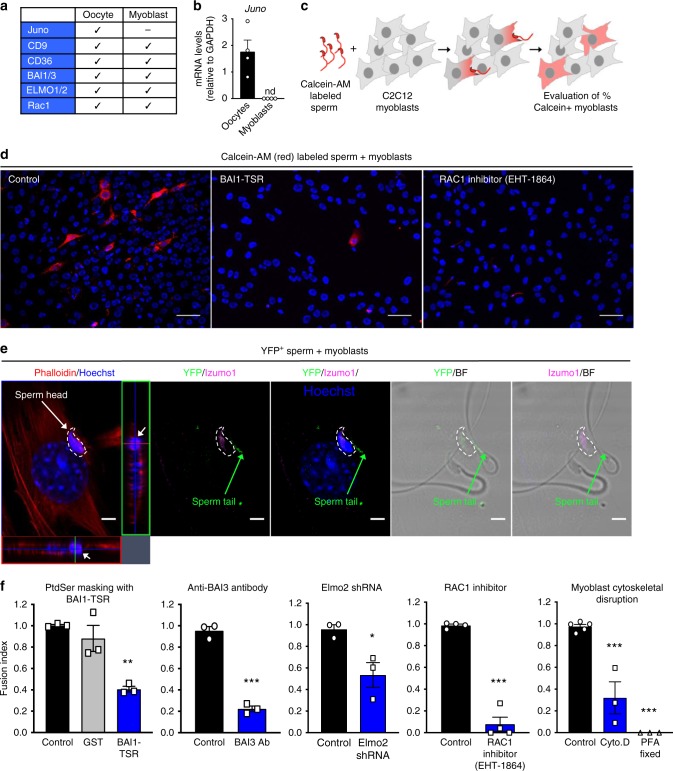
Sperm:myoblast fusion via PtdSer and the BAI3-ELMO2-RAC1 signaling axis. **a** Oocytes and myoblasts express similar molecules. Juno expression is readily detected on oocytes but not myoblasts. Bars represent mean ± s.e.m. **c**, Schematic of the sperm:myoblast fusion assay. Caudal epididymal sperm were labeled with Calcein-AM (red) and co-cultured with murine C2C12 myoblasts. After 4 h, myoblasts were washed, stained with Hoechst dye (to stain nuclei) and the percentage of Calcein-AM^+^ myoblasts was evaluated by microscopy. **d** Representative images depicting myoblasts that fuse with sperm to become Calcein-AM^+^ (3–10% per field) under control conditions (left panel), whereas this is greatly reduced when the sperm was pretreated with BAI1-TSR to mask PtdSer (middle panel) or after treatment of the myoblasts with the RAC1 inhibitor (right panel). Scale bar, 50 μm. **e** Detection of sperm inside the myoblasts. YFP^+^ sperm were co-incubated with C2C12 myoblasts for 4 h. Myoblasts were washed, fixed, and stained by immunofluorescence with antibodies to YFP/GFP (green) and Izumo1 (pink). Phalloidin (red) and Hoechst (blue) were used to stain the actin cytoskeleton and DNA, respectively. On the left panel, the dense sperm nucleus contained within the phalloidin^+^ cytoplasm is shown on the cross-sectional plane. White arrows: sperm nucleus; green arrow: sperm tail; dotted line: outline of the sperm head. Scale bar: 5 μm. **f** Reduced percentage of Calcein-AM^+^ myoblasts after masking of PtdSer on sperm using BAI1-TSR, antibody to BAI3, shRNA-mediated knockdown of *Elmo2* in myoblasts, treatment of myoblasts with the RAC1 inhibitor EHT-1864 or after pretreatment of myoblasts with cytoskeletal disruption via cytochalasin D (Cyto. D) or paraformaldehyde (PFA) fixation. (*n* = 3 independent experiments for each condition except for RAC1 inhibitor, *n* = 4 independent experiments). Bar charts show mean ± s.e.m. *p < 0.05, **p < 0.001, ***p < 0.0001 (one-way ANOVA followed by Dunnet’s multiple comparisons test and two-tailed unpaired Student’s *t* test). Each dot represents one experiment. Source Data are provided in the Source Data File

Our results up to this point suggest that PtdSer on the sperm and its receptors BAI1/3, CD36, Tim-4, and Mer-TK on the oocyte can promote fusion via the ELMO–RAC1 signaling pathway. Our laboratory and others^19,25,49^ have previously demonstrated that PtdSer exposure on skeletal myoblasts is important for the fusion between myoblasts to form myotubes, and this occurs in a BAI1/3–ELMO–RAC1-dependent manner^19,25,49,55^. Intriguingly, when we examined the expression of genes linked to the sperm:egg fusion in myoblasts, we found that oocytes and myoblasts both expressed the membrane proteins CD9, CD36, BAI1, and BAI3, as well as cytoplasmic ELMO2, and RAC1 (Fig. 4a)^8,25,49,56–59^, whereas Juno expression was not detected in myoblasts (Fig. 4b). Therefore, we asked whether caudal sperm could fuse with skeletal myoblasts, as it has been shown with other somatic cells^60,61^, and whether this Juno-independent fusion was mediated by the BAI1-ELMO1-RAC1 module expressed by myoblasts.

We labeled caudal sperm with a red-fluorescent cytoplasmic dye (Calcein-AM)^60^, incubated them with C2C12 mouse myoblasts, and looked for myoblasts that acquire the sperm-derived Calcein-AM staining (Fig. 4c). Of note, there was no myoblast:myoblast fusion when they were in growth medium in a non-confluent state. However, some of these myoblasts are known to be poised for fusion^25^. Remarkably, we could readily detect transfer of sperm-derived Calcein-AM into few of the myoblasts in a quantifiable manner (Fig. 4d). To further address the fusion between sperm and skeletal myoblasts, we took three additional approaches. First, labeling sperm with another dye (DiI) produced similar results as scored by fusion with myoblasts (Supplementary Fig. 4). Second, we isolated sperm from mice expressing transgenic yellow fluorescent protein (YFP) and, after incubating them with myoblasts, we detected for YFP/GFP (green fluorescent protein) and the sperm-specific protein Izumo1 within the fusing myoblasts by immunostaining. We could readily observe the signal for YFP, and the sperm specific protein Izumo1, in addition to the DNA from the sperm head within the myoblasts (Fig. 4e). As a third approach, we used electron microscopy to detect the presence of sperm within myoblasts. The midpieces (containing multiple mitochondria), and the tails from multiple sperm could be detected inside the cytoplasm of a myoblast (Supplementary Fig. 5c). Of note, we do not detect any obvious membrane surrounding the sperm structures, suggesting that the sperm is not contained within a phagocytic vesicle. Interestingly, aminophospholipid asymmetry on myoblasts differs from fibroblasts^16^, and this may, in part, explain sperm fusion with myoblasts but not fibroblasts (not shown). As C2C12 myoblasts are an immortalized cell line, we also tested whether sperm could fuse with cultured mouse primary myoblasts, which also express BAI1/3 and CD36 (Supplementary Fig. 6a), and this was indeed the case (Supplementary Fig. 6b, c). Further, when we incubated sperm with primary bone marrow- derived macrophages, the sperm-derived Calcein-AM was not dispersed within the cytoplasm of macrophages as was the case with myoblasts, but rather the sperm appeared to be phagocytosed by the macrophages (Supplementary Fig. 7).

We next asked whether this sperm:myoblast fusion event also depends on PtdSer exposure on the sperm and the BAI1-ELMO–RAC1 module on the myoblast. First, blocking PtdSer on the sperm (via BAI1-TSR) significantly decreased the fusion of sperm to myoblasts (Fig. 4d, f). Second, antibody-mediated blocking of BAI proteins [BAI3 is expressed at a much higher level than BAI1 in C2C12 myoblasts^49^) potently blocked sperm fusion to myoblasts (Fig. 4f). Third, C2C12 myoblasts with knockdown of *Elmo2* [the predominant ELMO isoform expressed in C2C12 myoblasts^49^] showed a significantly reduced Calcein-AM acquisition from the labeled sperm (Fig. 4f). Fourth, the RAC1 inhibitor EHT-1864 also potently blocked the sperm:myoblast fusion (Fig. 4d, f and Supplementary Fig. 4). Consistent with the BAI1/3–ELMO–RAC1 module being involved in cytoskeletal rearrangements in cell:cell fusion during myotube formation, the treatment of myoblasts with cytochalasin D, or fixation of myoblasts with paraformaldehyde, prior to adding the sperm abrogated the sperm-derived fluorescence acquisition by myoblasts (Fig. 4f and Supplementary Fig. 4). These data suggest that PtdSer exposure on viable sperm and its interaction via the BAI3-ELMO–RAC1 module on myoblasts can contribute to cell:cell fusion, in a Juno-independent manner. As both myoblast:myoblast^56^ and myoblast:sperm fusion (Supplementary Fig. 8) are also dependent on CD9, another surface molecule relevant in sperm:egg fusion^8,62^; it is possible that PtdSer receptors may work together with CD9 during sperm:myoblast fusion. Although this system scores fusion between a diploid myoblast and a haploid sperm (i.e., non-productive), this could potentially serve as an assay to test sperm competency among infertile couples. Moreover, C2C12 myoblasts are easier to manipulate and genetically modify compared to oocytes, and may provide a good system to probe the mechanistic aspects of sperm fusion.

## Discussion

Taken together, the data presented in this work provide several insights relevant for sperm:egg fusion during mammalian fertilization. Using different approaches our data demonstrate that the heads of viable and motile sperm display PtdSer. PtdSer asymmetry on the leaflets of plasma membrane is orchestrated in a complex way, via multiple proteins functioning in opposite directions; further, PtdSer is also essential for many intracellular trafficking events. Deletion of genes coding for enzymes that are required for PtdSer synthesis, *Ptdss-1* and *Ptdss-2*, leads to embryonic lethality^63^. As mice for conditional targeting of *Ptdss-1* or *Ptdss-2* genes are not currently available, we took the approach of masking or competitively interfering with the PtdSer on sperm. This led to reduction in in vitro fertilization ranging from 40 to 85% with three different PtdSer-targeting agents (including Annexin V, the highest affinity reagent, with the best effect). From the oocyte end, blocking of individual PtdSer receptors in isolation often had modest effects, likely owing to the well-known redundancy among PtdSer receptors in phagocytosis studies; however, combinatorial targeting multiple PtdSer receptors via antibody-mediated blocking, genetic deletions, and disruption of cytoplasmic signaling approaches suggest a role for the PtdSer receptors in sperm entry into oocytes. Mechanistically, analogous to the role of PtdSer and its receptors in cell:cell fusion in other systems, our data suggest that PtdSer on sperm, PtdSer receptors and the downstream signaling molecules in the oocyte can have a role in sperm:egg fusion.

We also acknowledge a limitation of our study. In the field of fertilization, ablation of in vivo fertility after genetic deletion has become the gold standard to demonstrate the requirement for molecules critical in fertilization. This has been only been met for a few genes coding for proteins such as Izumo1, Juno, and CD9. This criterion was extremely hard to meet in our study because PtdSer is a lipid on cell membranes, and consistent with the myriad roles in normal cellular functions, and the complex number of steps that control PtdSer asymmetry, genetically achieving a PtdSer-null mature sperm (expecting to develop normally through the various steps of spermatogenesis) is currently unfeasible. As PtdSer receptors are also highly redundant, generation of mice lacking 3, 4, or 5 different PtdSer also poses problems. Thus, the technical issue of genetically manipulating PtdSer exposure and its recognition remains to be tackled in the future. To assuage this issue, we have tested the roles of both PtdSer and its receptors from both the sperm and the oocytes sides, using multiple in vitro approaches (in contrast to previous works, where either ligands on the sperm or the oocyte were studied independently). Importantly, after blocking either the ligand on the sperm or the receptors/signaling pathway on the oocytes, we observed similar outcomes.

Synthesizing prior work and this report, our current model is as follows. We posit that Izumo1 and PtdSer on sperm could function cooperatively in mediating sperm binding to oocytes and fusion. This is based on the known properties of both PtdSer and Izumo1. When PtdSer acts as an eat-me signal or as a fusion signal, it is not sufficient by itself, and requires additional players for its functionality^45,64^. Further, caudal sperm already exposes PtdSer, whereas Izumo1 is exposed on caudal sperm after the acrosome reaction^4^; thus, both Izumo1 and PtdSer can be present on the sperm surface at the same time for subsequent interactions with the oocyte. We also envision multimeric interactions between the sperm and egg surfaces for achieving sufficient specificity and avidity. For example, Izumo1 is known to dimerize^7,65^; similarly, PtdSer binding often involves multiple receptors and cooperative polyvalent binding^40,64^. Based on the critical requirement for Izumo1:Juno interaction, we anticipate that this may provide the initial strong gamete binding, followed by the interaction between PtdSer on sperm and the PtdSer receptors on oocytes. At this point, the BAI1/3–ELMO–RAC1 module, along with other molecules that have been previously linked to fertilization on the oocytes (such as CD9), may help achieve gamete fusion. These hierarchical/step-wise interactions between the gametes, leading to fusion are in part supported by the sperm fusion with myoblasts (that are poised for fusion) in the absence of Juno. This type of combinatorial use of diverse molecules to achieve cell:cell binding/subsequent signaling is very analogous to interactions within the immune system: e.g., during T-cell interaction with an antigen-presenting cell, the central driver is the TCR:MHC/peptide interaction; yet, a host of other accessory molecules are fundamentally important for achieving specificity and optimal activation of T cells after antigen recognition. Similar to Juno (which is GPI-anchored and does not have its own signaling motif), the TCR does not have a signaling motif of its own and requires associated molecules for inducing downstream signaling and activation. Analogously, the PtdSer recognition receptors on the oocytes may work together with Juno in initiating intracellular signaling within oocytes, eventually leading to the critical step of gamete fusion. In summary, these data suggest that PtdSer on sperm and its receptors on oocytes as functional players that can work in conjunction with Izumo1 and Juno to promote sperm:egg fusion during fertilization.

## Methods

### Mice

C57BL/6 mice (stock 000664) and Ddx4-Cre mice (stock 006974) were purchased from the Jackson Laboratory and bred in our facilities. BAI1 deficient and Elmo1^fl/fl^ mice were previously generated in our laboratory^11^. Mer-tk-deficient mice (stock 011122) were purchased from the Jackson Laboratory and crossed with BAI1 deficient mice in our facilities. Tim-4-deficient mice were kindly provided by Dr. Vijay Kuchroo (Brigham and Women’s Hospital, MA). Yellow fluorescent protein (YFP) expressing mice (stock 006148) were crossed to E2A-Cre mice (stock 003724), both from the Jackson Laboratory. All animal procedures were approved by and performed according to the guidelines of the Institutional Animal Care and Use Committee (IACUC) at the University of Virginia.

### Sperm staining

To stain sperm with Annexin V, the caput, corpus, and cauda epididymis of adult (> 10 weeks old) male mice were dissected and the sperm were allowed to disperse for 15 min in capacitating medium [TYH + bovine serum albumin (BSA) (119 mm NaCl, 4.7 mm KCl, 1.71 mm CaCl_2_, 1.2 mm KH_2_PO_4_, 25.1 mm NaHCO_3_, 5.56 mm glucose, 0.51 mm Na pyruvate, 1% phenol red, supplemented with 4 mg/ml BSA, penicillin, and streptomycin)^66^]. For capacitation studies, caudal sperm were allowed to swim in capacitating medium (described above) or non-capacitating medium (lacking CaCl_2_, NaHCO_3_ (replaced with 4-(2-hydroxyethyl)-1-piperazineethanesulfonic acid) and 4 mg/ml BSA) and incubated in the respective medium for additional 90 min. Sperm were counted and 1 × 10^6^ cells were washed in 1×binding buffer and stained with Alexa-Fluor 568 conjugated Annexin V (Life Technologies) for 15 min. After washing with binding buffer and staining with Hoechst, sperm were placed on slides and 6–8 photographs were taken per sample. The percentage of Annexin V + sperm (stained on head, midpiece, or midpiece and head) was calculated.

To visualize the motility of Annexin V^+^ sperm, caudal sperm were obtained as described above, capacitated for 90 min in TYH + BSA at 37 °C, 5% CO_2_, and stained with Annexin V conjugated with Alexa-Fluor 488 (Life Technologies) for 15 min at room temperature. Sperm aliquots were placed on 20 μm chamber slides (Leja) and analyzed in a Ziess LSM880 confocal microscope (Microscope Facility Core, University of Virginia). To detect PtdSer, sperm was also incubated with BAI1-TSR-GST or GST only (50 μg/ml), washed and fixed with 2% paraformaldehyde and placed on slides. GST was detected with a specific antibody (GST Ab, Santa Cruz, sc-138B) followed by a biotinylated secondary antibody and NA-Texas Red.

To test the staining sperm with Duramycin, sperm from the cauda epididymides were obtained as described above, incubated with Duramycin-LC-biotin conjugate (Molecular Targeting Technologies), washed and stained with NA-Texas Red (Southern Biotech). After an additional wash, sperm were stained with Hoechst and mounted in coverslips.

To stain sperm with the dyes Calcein-AM or DiI, caudal sperm were allowed to swim in TYH + 0.75 mm Methyl-beta-cyclodextrin (MBCD)^67^ for 15 min, counted and stained with 4 μm Calcein red orange-AM (Invitrogen) for 30 min in PBS at room temperature or with 5 μg/ml DiI (Invitrogen) for 30 min at 37 °C in HBSS, as previously described^60^. Cells were washed, counted and resuspended in Dulbecco's modified Eagle's medium (DMEM) medium supplemented with 20% heat inactivated FBS.

### In vitro fertilization (IVF) assays

IVF was performed as previously described^68^. In brief, 4–5 weeks old female mice were super-ovulated by intraperitoneal injections of 5 IU of pregnant mare serum gonadotropin (Prospec) and 5 IU of human chorionic gonadotropin (hCG, Sigma) 48 h later. Metaphase II oocytes were recovered 13 h after hCG, and were: (1) directly used as cumulus oocyte complexes, (2) freed from cumulus cells by a brief incubation with 3 mg/ml hyaluronidase (Sigma) in 0.01% poly vinyl alcohol/FHM medium (EMD Millipore). These oocytes were labeled as ZP-intact oocytes; or (3) freed from cumulus cells and ZP using Tyrode’s solution (Sigma) (ZP-free oocytes). Sperm were collected from the cauda epididymides of adult (> 10 week old) wild-type male mice and capacitated for 90 min in TYH + BSA medium (described above, sperm staining section). All the incubations were performed in medium drops under paraffin oil (Sigma) at 37 °C, 5% CO_2_ atmosphere.

To evaluate the role of PtdSer exposed on sperm, cumulus oocyte complexes (*n* = 30–60) were inseminated with 2–4 × 10^4^ sperm capacitated for 60–90 min in TYH + BSA (in most of the experiments) or in TYH + MBCD^67^ (in the experiment to test the effect of Annexin V in fertilization, Fig. 1k). During the last 30 min of capacitation, sperm were incubated with 10 μg/ml Annexin V (Ebiosciences), 50 μg/ml GST or 50 μg/ml GST-BAI1-TSR. After co-culture of oocytes and sperm in 100 μl drops of high calcium human tubal fluid supplemented with 1 mm reduced glutathione medium for 4 h^67^, eggs were washed several times and transferred to KSOM medium (EMD Millipore). The percentage of two-cell embryos (fertilized eggs) was determined at 24 h post insemination. Sperm motility upon incubation with control medium, Annexin V, GST, or GST-BAI1-TSR was graded as progressive motility, non-progressive motility, and immotility, according to the criteria of the 5 Edition of the World Health Organization (WHO) Laboratory Manual for the Examination and Processing of Human Semen. The non-progressive motility and immotility numbers were grouped together. To test the soluble head group of PtdSer, *O*-Phospho-l-Serine (Sigma) and its control *O*-Phospho-d-Serine (Sigma) in IVF assays, ~ 20 ZP-free oocytes isolated from wt mice were incubated 1 × 10^3^ capacitated sperm in 20-μl droplets for 1 h in the presence of 1 mm *O*-Phospho-l-Serine or *o*-Phospho-d-Serine. After washes and an overnight incubation the percentage of two-cell embryos was evaluated.

To genetically test the role of PtdSer recognition receptors in the fertilization assays, BAI1^*−/−*^, ELMO1^*−/−*^, Tim-4^*−/−*^, Mer-tk^*−/−*^, BAI1^*−/−*^, and wild-type female mice were super-ovulated as described above and cumulus oocytes complexes were inseminated with 2 × 10^5^ capacitated sperm in 100 μl drops of TYH + BSA for 4 h. After several washes, oocytes were transferred to KSOM medium and the percentage of two-cell embryos was evaluated at 24 h post insemination.

To evaluate the role of BAI1/3 and CD36 using blocking antibodies (Abs), we incubated cumulus-free ZP-intact oocytes with BAI1/3 (50 μg/ml, R&D Systems, AF4969), CD36 (10 μg/ml, clone CFR D-2712, Hycult, HM1074), Juno (10 μg/ml, clone TH6, Biolegend, 125102), CD9 (50 μg/ml, clone KMC8, BD Biosciences, 553758) antibodies or isotype controls: mouse IgA, (10 μg/ml, Southern Biotechnologies, 0106-14) or sheep IgG, (50 μg/ml, R&D systems, 5-001-A) in TYH + BSA + 5% FBS (to avoid ZP hardening) for 1 h at 37 °C, 5% CO_2_. After several washes in TYH + BSA (to eliminate the FBS) 25–30 oocytes were inseminated with 4 × 10^4^ capacitated sperm in 100 μl TYH + BSA in the presence or absence of specific antibodies or isotype controls for 3 h. Oocytes were washed in TYH + BSA and transferred to KSOM medium for an overnight incubation at 37 °C, 5% CO_2_. The fertilization index was calculated as the percentage of fertilized eggs (two-cell embryos) in the experimental group divided the percentage of fertilized eggs observed in the control group.

To score fertilization via the sperm DNA decondensation assay, we incubated cumulus-free ZP-intact oocytes with CD36, BAI1/3, or the isotype controls as described above, and then loaded the oocytes with 10 μg/ml DAPI (BioRad) for 20 min at 37 °C, 5% CO_2_, and washed several times in TYH + BSA^3^. Oocytes were inseminated for 3 h, washed and fixed with 0.25% glutaraldehyde and 0.1% paraformaldehyde for 20 min at room temperature. The percentage of oocytes with DAPI^+^ decondensed sperm nuclei (typically 1–2/oocyte) was evaluated by microscopy.

To evaluate the role of RAC1, ZP-free oocytes were prepared as described above, loaded with 10 μg/ml DAPI (BioRad) for 20 min at 37 °C, 5% CO_2_, and washed several times in TYH + BSA^3^. Twenty oocytes, either untreated or previously incubated with RAC1 inhibitor (80 μm, EHT-1864, Sigma) for 1–2 h were inseminated with 1000 capacitated sperm for 1 h in 20 μl drops of TYH + BSA medium at 37 °C, 5% CO_2_. RAC1 inhibitor was also present the fertilization steps. After three washes, oocytes were fixed in 0.25% glutaraldehyde and 0.1% paraformaldehyde for 20 min at room temperature, washed and mounted. The percentage of oocytes with DAPI^+^ decondensed sperm nuclei (typically 1–2/oocyte) was evaluated by microscopy.

### Staining of mouse/human eggs with antibodies

ZP-free oocytes were obtained from wild-type female mice after removal of cumulus cells and the ZP via treatment with hyaluronidase and Tyrode’s solution, respectively. Live oocytes were incubated with antibodies to BAI1/3 (R&D Systems), CD36 (Hycult), CD9 (BD Pharmingen, 553758), Juno (Biolegend, 125102), or both CD36 + BAI1/3 Abs in TYH + BSA drops under paraffin oil at 37**°**C, 5% CO_2_ for 1 h. Antibodies concentrations were the same as described above (IVF section). For the staining of microvilli, oocytes were incubated with 50 μg/ml fluorescein isothiocyanate-Concanavalin A^37^ (Vector, FL-1001) for 5 min. Cells were fixed in 4% paraformaldehyde (PFA) + 1% BSA for 30 min at room temperature, washed and incubated with biotinylated secondary or Alexa-Fluor 488 secondary antibodies for 1 h at room temperature. After washing, cells were stained with NA-Texas Red and Hoechst in 1% BSA. For ELMO1 staining, oocytes were fixed in 4% PFA + 1% BSA and permeabilized with 0.5% triton X-100 + 5% BSA and the stained with an ELMO1 antibody (Abcam, ab2239) overnight at 4 °C. ZP-intact human oocytes were stained with BAI1/3 or CD36 antibodies and fixed as described with mouse oocytes. Informed consent was obtained from female IVF patients undergoing treatment at the Reproductive Medicine and Surgery Center of Virginia to use discarded, de-identified unfertilized human oocytes following approval by the Sentara Martha Jefferson Hospital Institutional Review Board (IRB #06-017).

The presence of functional PtdSer receptors on mouse ZP-free wild-type oocytes was evaluated by incubation with 2 μm carboxylate modified red (580/605) beads (1:400, Invitrogen, FluoSpheres) for 2 h in TYH + BSA at 37 °C, 5% CO_2_. Beads were pretreated with medium only, 50 μg/ml GST or 50 μg/ml GST-BAI1-TSR for 30 min. Oocytes were washed six times in TYH + BSA, stained with CD9 antibody for 30 min at 37 °C, 5% CO_2_ in TYH + BSA, fixed in 4% PFA + 1% BSA and stained with Hoechst. The percentage of oocytes with bound beads was determined by microscopy. We scored oocytes with > 5 bound beads, as accurately counting individual beads, particularly in oocytes of the control groups that bound multiple beads was unfeasible.

### Immunostaining of ovarian tissue

Mouse ovaries obtained from 4–6 week-old wild-type female mice were fixed in Bouin’s solution for 6 h and embedded in paraffin (Histology Core, University of Virginia). Sections were deparaffinized and hydrated, and stained with BAI1/3, Connexin-43 (Abcam, ab11370), or ELMO1 antibodies as described above for isolated oocytes. Human ovary was stained with BAI1/3 Ab (R&D Systems) by the Biorepository and Tissue Research Facility at the University of Virginia.

### Sperm:myoblast fusion experiments

C2C12 murine skeletal muscle myoblasts (ATCC) were maintained at sub-confluent densities in DMEM medium supplemented with 20% heat inactivated FBS at 37 °C, 8.5% CO_2_. Cells were trypsinized, counted and plated in four-well LabTek II Permanox chamber slides (25,000 cells/well). After 24 h, myoblasts were treated with appropriate buffer alone, or 5 μg/ml Cytochalasin D (Sigma), 80 μm RAC1 inhibitor (EHT-1864), or incubated with Abs (BAI3 [R&D Systems, MAB39651] or CD9 [BD Biosciences]) for 30 min at 37 °C, 8.5% CO_2_ prior to the sperm co-incubation. Alternatively, myoblasts were fixed with 4% PFA for 20 min and washed several times. Calcein-AM + sperm (250,000 sperm/well) were added and incubated for 4 h at 37 °C, 8.5% CO_2_. The blocking agents were also present during the co-incubation except for Cytochalasin D, which was used only during the preincubation to avoid affecting the sperm. Sperm were pretreated with 50 μg/ml GST or 50 μg/ml GST-BAI1-TSR for 30 min and then added to the myoblasts. C2C12 myoblasts with shRNA-mediated *Elmo2* knockdown have been described previously^25^. After the 4 h co-incubation, myoblasts were washed, stained with Hoechst and mounted. Cells were analyzed the same day and 6–8 fields/well were photographed using the Axio Imager 2 with Apotome (Zeiss). Image J cell counter plugin software was used to quantitate nuclei number. The fusion index was calculated as the percentage of Calcein-AM^+^ myoblasts in the experimental group normalized to the control untreated cells.

Primary myoblasts were isolated from 3–6 weeks old mice as previously described^69,70^ and plated in Matrigel-coated dishes. Detection of BAI1/3, CD36 and CD9 was performed by immunofluorescence as described above. For the detection of YFP sperm within myoblasts, YFP + sperm was co-cultured with C2C12 myoblasts for 4 h as described above, and fixed in 3.7% formaldehyde for 5 min at room temperature. Cells were permeabilized with 0.5% Triton X-100 for 10 min and incubated overnight at 4 °C with antibodies to GFP/YFP (Abcam, ab6673) and Izumo1 (ProSci, 8233) diluted in 0.1% Tween-20 and 0.5% BSA. Secondary antibodies conjugated with Alexa-Fluor 647 or biotin were used. Streptavidin–-Texas Red, Phalloidin (Invitrogen) and Hoechst were also used. Myoblasts were analyzed with a LSM880 confocal microscope. The YFP/GFP antibody staining was necessary as the endogenous fluorescence of YFP was too weak to detect after fixation. For electron microscopy, unlabeled sperm were co-cultured with C2C12 myoblasts for 4 h. Cells were washed to remove the residual unfused sperm, trypsinized, and washed again before fixation with 2.5% glutaraldehyde and 2% paraformaldehyde, and processing for analysis via electron microscopy.

### Quantitative RT-PCR

Total RNA was extracted from cumulus-free ZP-intact oocytes from wild-type female mice using Trizol (Ambion) and the cDNA was synthesized using QuantiTect Reverse Transcription Kit (Qiagen) according to manufacturer’s instructions. qPCR for mouse *Bai1, Bai2, Bai3, Tim-4, Stab2, Elmo1, Elmo2, cd36, cd52, Juno*, or housekeeping gene *Gapdh* was performed using Taqman probes (Applied Biosystems) using StepOnePlus Real Time PCR System (ABI). *Cd52* was used to determine oocyte contamination with cumulus cells.

### Statistical analysis

Statistical significance was determined using GraphPad Prism 5 or 6 using unpaired Student’s two-tailed *t* test, one-sample *t* test, Mann–Whitney test, or one-way analysis of variance as according to test requirements. No inclusion/exclusion criteria were pre-established. A *p* value of < 0.05 (*), < 0.01 (**), or < 0.001 (***) were considered significant.

## Supplementary information

Supplementary information

Description of Additional Supplementary Files

Supplementary movie 1

Source data file

## Data Availability

All the relevant data supporting the key findings of this study are available within the article and its Supplementary Information files or from the corresponding authors upon reasonable request. The source data underlying Figs. 1e, k, l, n, 2c, d, i, l, 3b, c, d, g, j, k, 4b, f, and Supplementary Figs. 1a, 1e, 1f, 3f, 3d, 3e, 4d, 8b are provided as a Source Data file. A reporting summary for this Article is available as a Supplementary Information file.
