# Supplementary material for: Phosphatidylserine on viable sperm and phagocytic machinery in oocytes regulate mammalian fertilization
Source: Nat Commun. 2019 Oct 1;10:4456. doi: 10.1038/s41467-019-12406-z (PMC6773685; doi:10.1038/s41467-019-12406-z)
Supplement: Supplementary file 1 — Supplementary information [file 41467_2019_12406_MOESM1_ESM.pdf]

Supplementary Information

Phosphatidylserine on viable sperm and phagocytic machinery in oocytes regulate mammalian fertilization

Rival et al.

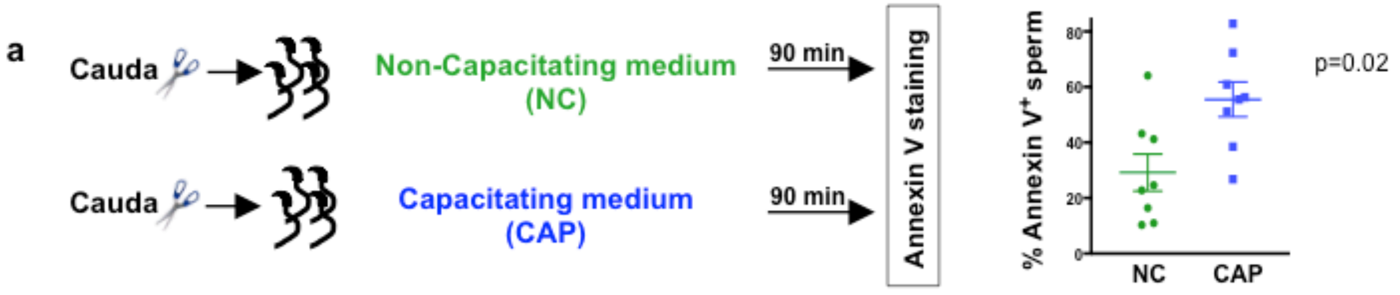

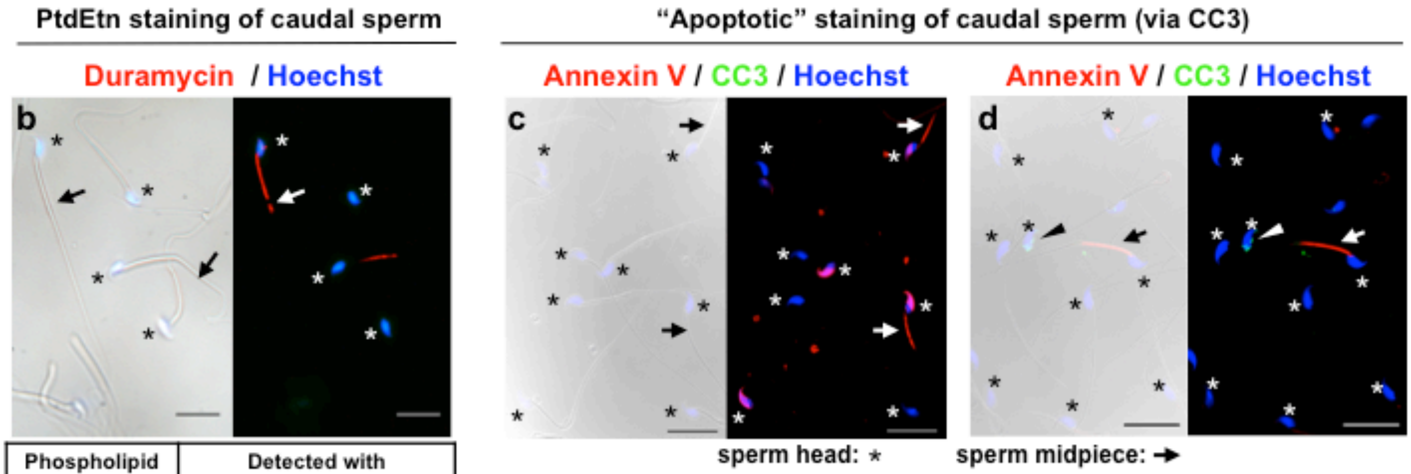

Phospholipid	Detected with
PtdSer	Annexin V / BAI1-TSR
PtdEtn	Annexin V / Duramycin

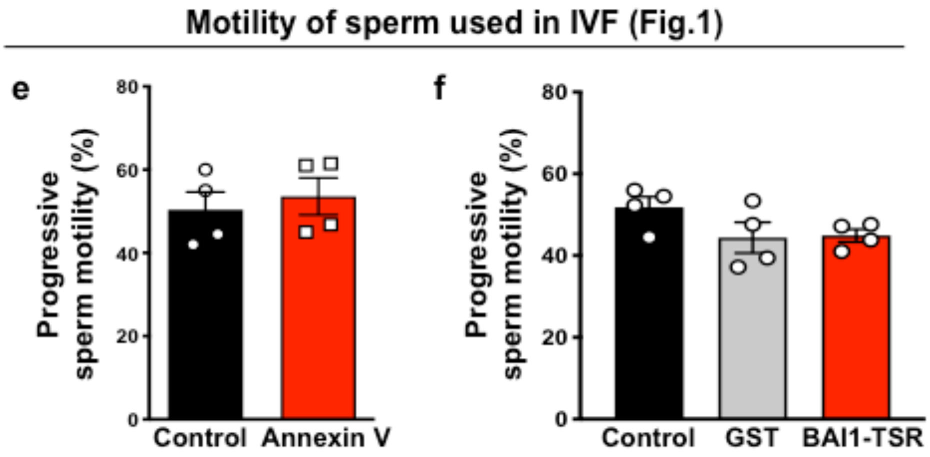

Supplementary Figure 1. Analysis of PtdSer exposure on sperm. a, PtdSer exposure on sperm surface is enhanced during capacitation. Experimental design (left) showing the isolation and incubation of caudal sperm in non-capacitating (top, green) or capacitating medium containing $CaCl_2$, BSA and $NaHCO_3$ (bottom, blue). In each mouse, one cauda (right) was used for the non-capacitating medium while the other cauda (left), was placed on capacitating medium. An increased percentage of Annexin V+ sperm is observed when capacitating medium was used (right). Each dot represents one mouse (n=8 mice) from 4 independent experiments. Mean ± s.e.m. *p=0.02 (Two tailed Mann Whitney non-parametric t-test). **b**, Phosphatidylethanolamine (PtdEtn) was stained in caudal sperm using biotinylated Duramycin followed by streptavidin-Texas Red. Asterisks point to sperm head and arrows denote Duramycin+ sperm midpieces. Scale bar, 20 µm. **c, d** "Fresh" caudal sperm (**c**) and sperm cultured for 24h (**d**) were stained with Annexin V (red), fixed, permeabilized and stained with a Cleaved Caspase 3 (CC3 Cell Signaling, 9661S) Ab (green) by immunofluorescence. **c**: No CC3+ sperm were detected. **d**: Arrowhead: CC3+ sperm. Asterisks point to sperm head and arrows to Annexin V+ sperm midpiece (**c** and **d**). Scale bar, 20 µm. **e, f**, To rule out effects on sperm vitality due to the buffers or the reagents used in *in vitro* fertilization assays (such as Annexin V, GST, or GST-BAI1-TSR), progressive motility was assessed. No significant differences were detected (n= 4 experiments). Bar charts show mean ± s.e.m. (**e**: p>0.05, two-tailed unpaired Student's t-test; **f**: p>0.05 one-way ANOVA). Source Data are provided in the Source Data File.

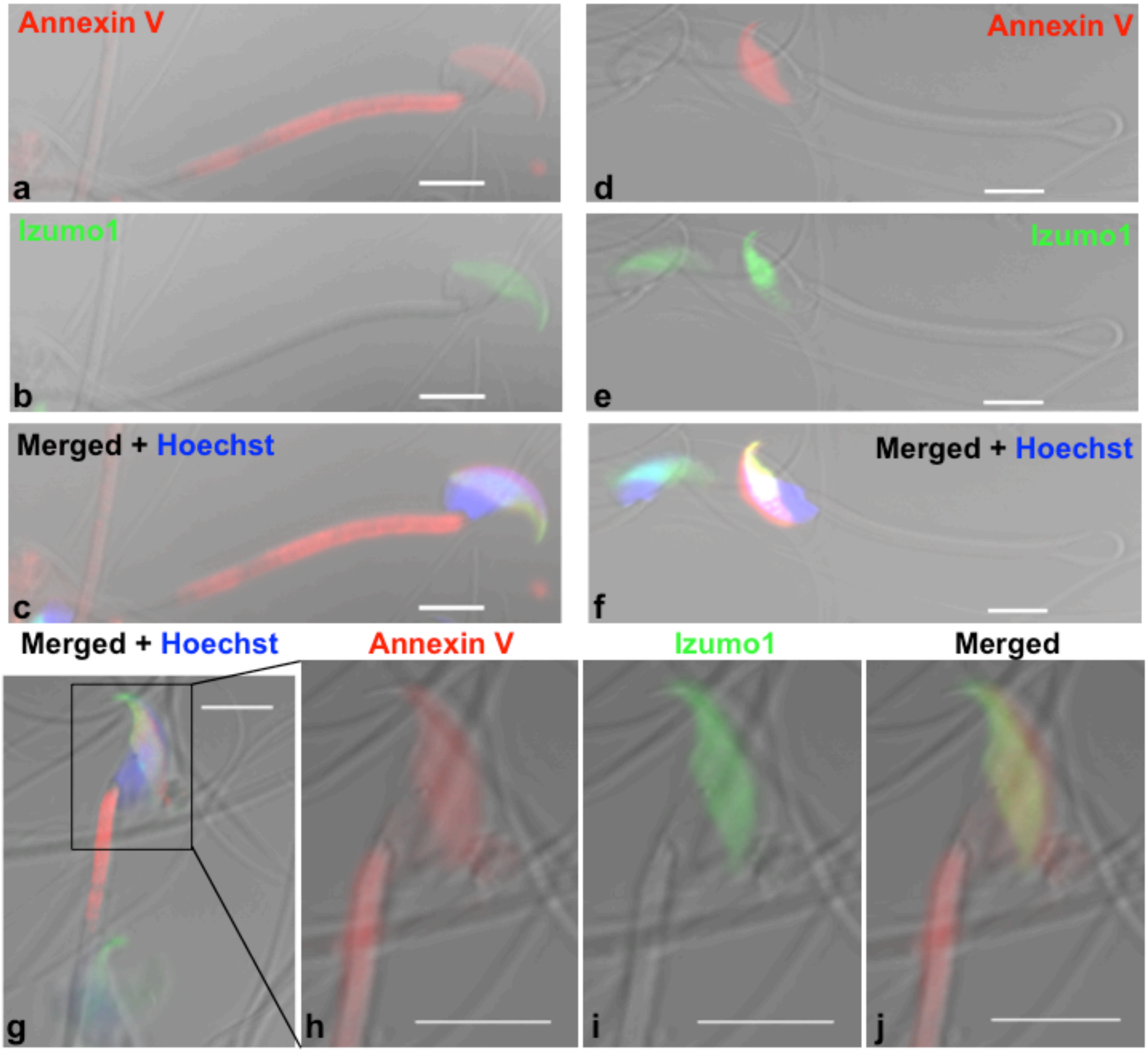
Annexin V
a
Izumo1
b
Merged + Hoechst
c
Annexin V
d
Izumo1
e
Merged + Hoechst
f
Merged + Hoechst
g
Annexin V
h
Izumo1
i
Merged
j

Supplementary Figure 2. Co-localization of PtdSer and Izumo1 on acrosome reacted sperm. Three different sperm are shown (**a-c; d-f; g-j**) with staining of PtdSer and Izumo1 on the equatorial region of the sperm head. **h-j**: higher magnification of boxed region in **g**. After capacitation of caudal sperm in TYH+BSA medium, the acrosome reaction was induced with the Ca^{+2} ionophore A23187. Live sperm were stained with anti Izumo1 polyclonal antibody (which is exposed after acrosome reaction; ProSci, 8233) and Annexin V (conjugated with Alexa Fluor 568; red). Sperm were washed and fixed with 4% paraformaldehyde and placed on slides. Izumo1 antibody was detected with an anti-rabbit IgG-Alexa Fluor 488 (green, Invitrogen, A21208). Hoechst was used to stain nuclei. Samples were analyzed in a LSM 700 Zeiss Confocal microscope. Bar: 5 μm.

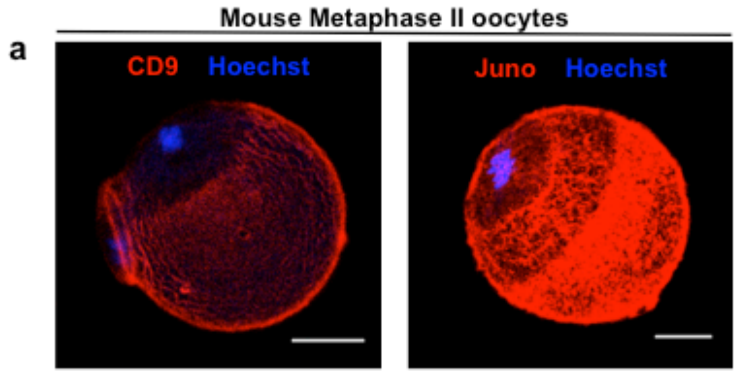

f

Treatment	% Fertilized eggs	# eggs
Untreated	81 ± 3.9%	224
Juno Ab	0.0 ± 0.0 %	58
CD9 Ab	27.3 ± 7.5 %	173

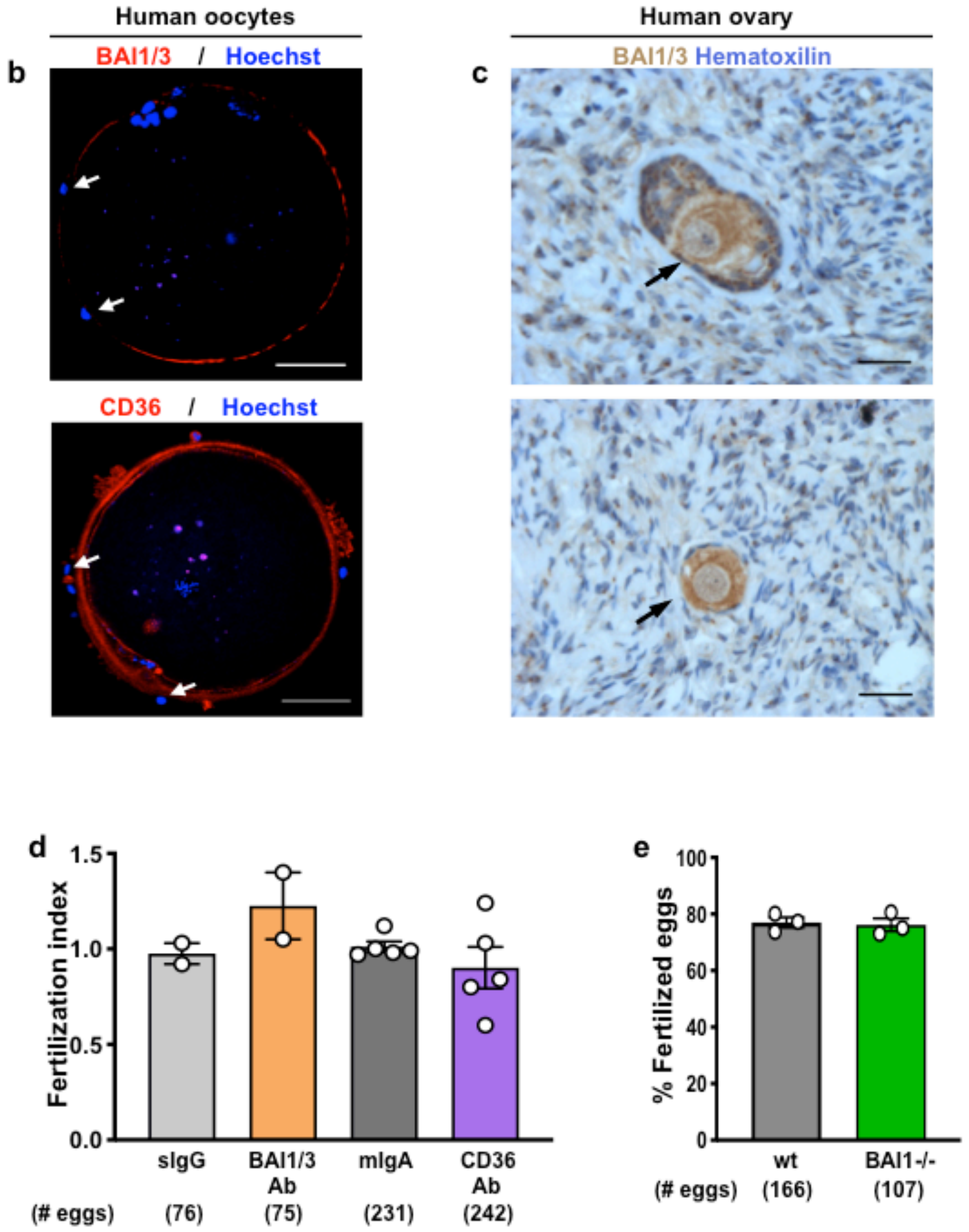

Supplementary Figure 3. Analysis of mouse and human oocytes. a, Staining of CD9 and Juno on mouse Metaphase II ZP-free oocytes. **b**, Staining of BAI1/3 (top) and CD36 (bottom) on human oocytes by immunofluorescence. Arrows indicate residual cumulus cells. Scale bar, 20 µm. **c**, Staining of BAI1/3 in a biopsy of human ovary by immunohistochemistry (arrows indicate oocytes). Scale bar, 50 µm. **d**, Antibodies targeting either BAI1/3 or CD36 alone do not affect fertilization. Fertilization index was calculated as percentage of fertilized eggs from the antibody treated group (isotype controls or CD36 or BAI1/3 antibodies) divided by percentage of fertilized eggs from the untreated group. Numbers in parentheses are total number of eggs from 2 independent experiments (sIgG and BAI1/3 Ab) and from 5 independent experiments (mIgA and CD36 Ab). Bar charts show mean ± s.e.m. sIgG: sheep IgG, mIgA: mouse IgA. ($p>0.05$; two-tailed unpaired Student's t-test). **e,** Oocytes from mice deficient in BAI1 show comparable fertilization to wt mice in the *in vitro* fertilization assays with wt sperm. Numbers in parentheses are total number of eggs, from 2 independent experiments (n=3 wt mice and n=3 BAI1-/- mice). Bar charts show mean ± s.e.m. ($p>0.05$; two-tailed unpaired Student's t-test). **f**, Fertilization is potently blocked with CD9 or Juno antibodies. ZP-intact oocytes from wt mice were pre-incubated with CD9 or Juno antibodies and inseminated with wt sperm (Juno Ab: n=2 independent experiments and CD9 Ab: n=3 independent experiments. After 24h, the percentage of fertilized eggs was evaluated (mean ± s.e.m). Source Data are provided in the Source Data File.

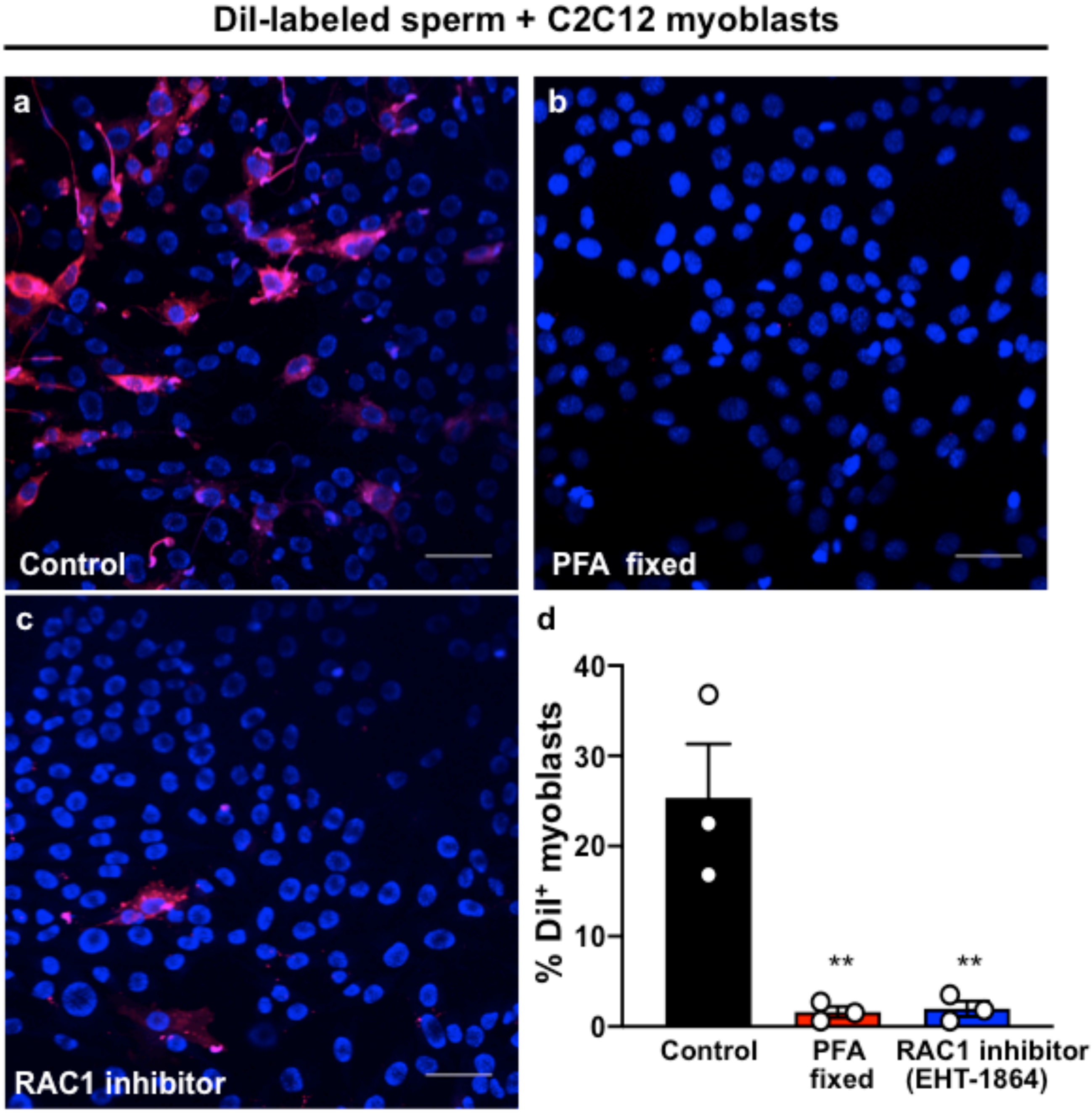

DiI-labeled sperm + C2C12 myoblasts
a
Control
b
PFA fixed
c
RAC1 inhibitor
d
% DiI+ myoblasts
40
30
20
10
0
**
**
Control
PFA fixed
RAC1 inhibitor (EHT-1864)

Supplementary Figure 4. Transfer of the dye DiI from the sperm to the myoblasts. a-c, C2C12 myoblasts were incubated with DiI-labeled sperm for 4h. After the co-culture, multiple DiI+ myoblasts were observed (**a**). This was blocked when myoblasts were fixed with paraformaldehyde (PFA) before co-incubation with sperm (**b**), or by the addition of the RAC1 inhibitor (EHT-1864) (**c**). Scale bar, 50 µm. **d,** The percentage of DiI+ myoblasts from 3 independent experiments is summarized. Bar charts show mean ± s.e.m. **$p<0.01$ (One-way ANOVA followed by Dunnet's multiple comparisons test). Source Data are provided in the Source Data File.

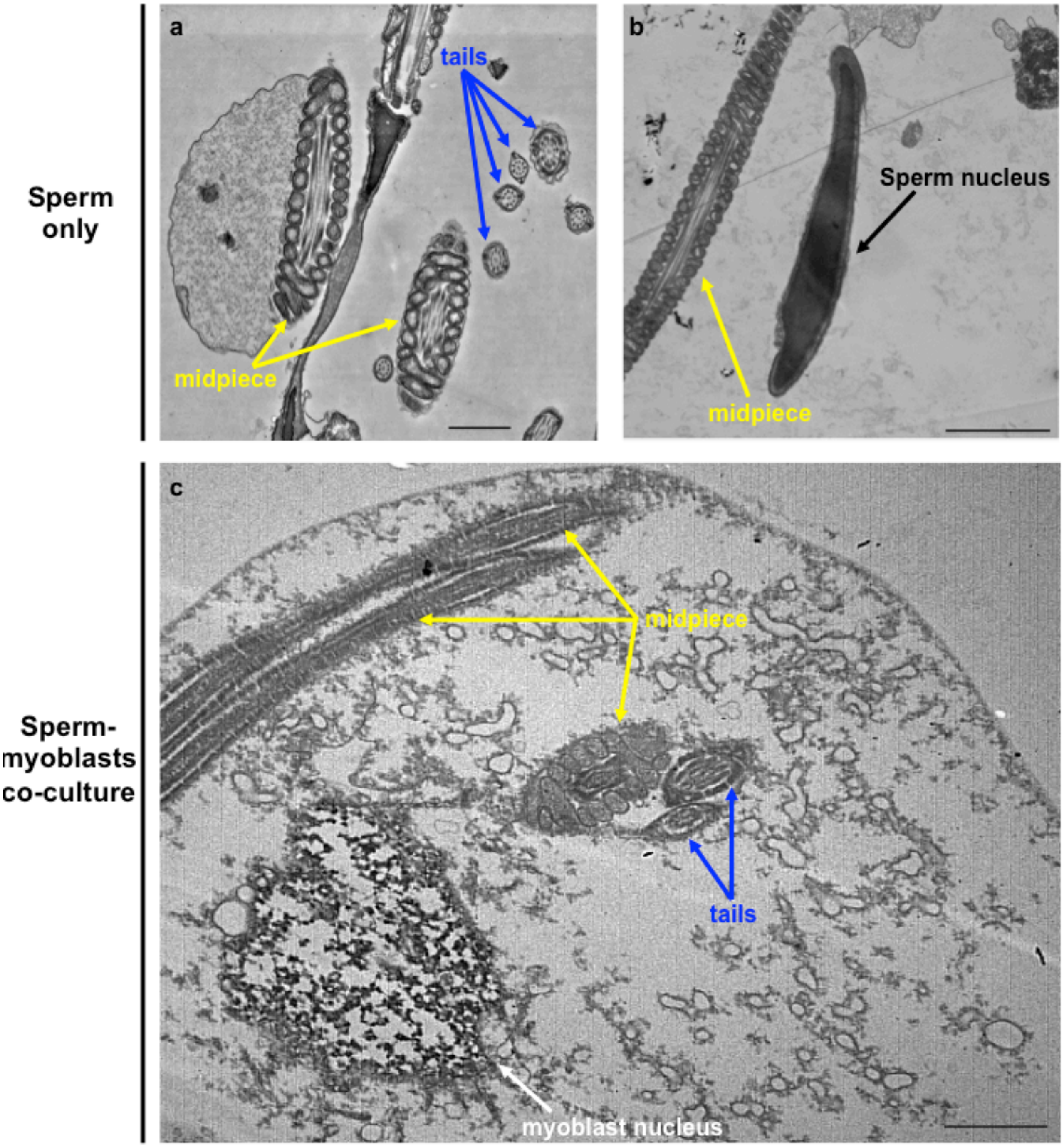
Sperm only
a
tails
midpiece
b
Sperm nucleus
midpiece
Sperm-myoblasts co-culture
c
midpiece
tails
myoblast nucleus

Supplementary Figure 5. Detection of sperm inside the myoblasts by electron microscopy. a-b, Electron microscopic images of sperm alone showing the typical structures of the midpiece with multiple mitochondria, the electron dense sperm nucleus, and the tail. Scale bar, 1 μm (**a**), 2 μm (**b**). **c**, After co-incubation of sperm with C2C12 myoblasts, the midpiece and tails of several sperm can be observed inside the myoblasts. Scale bar, 1 μm.

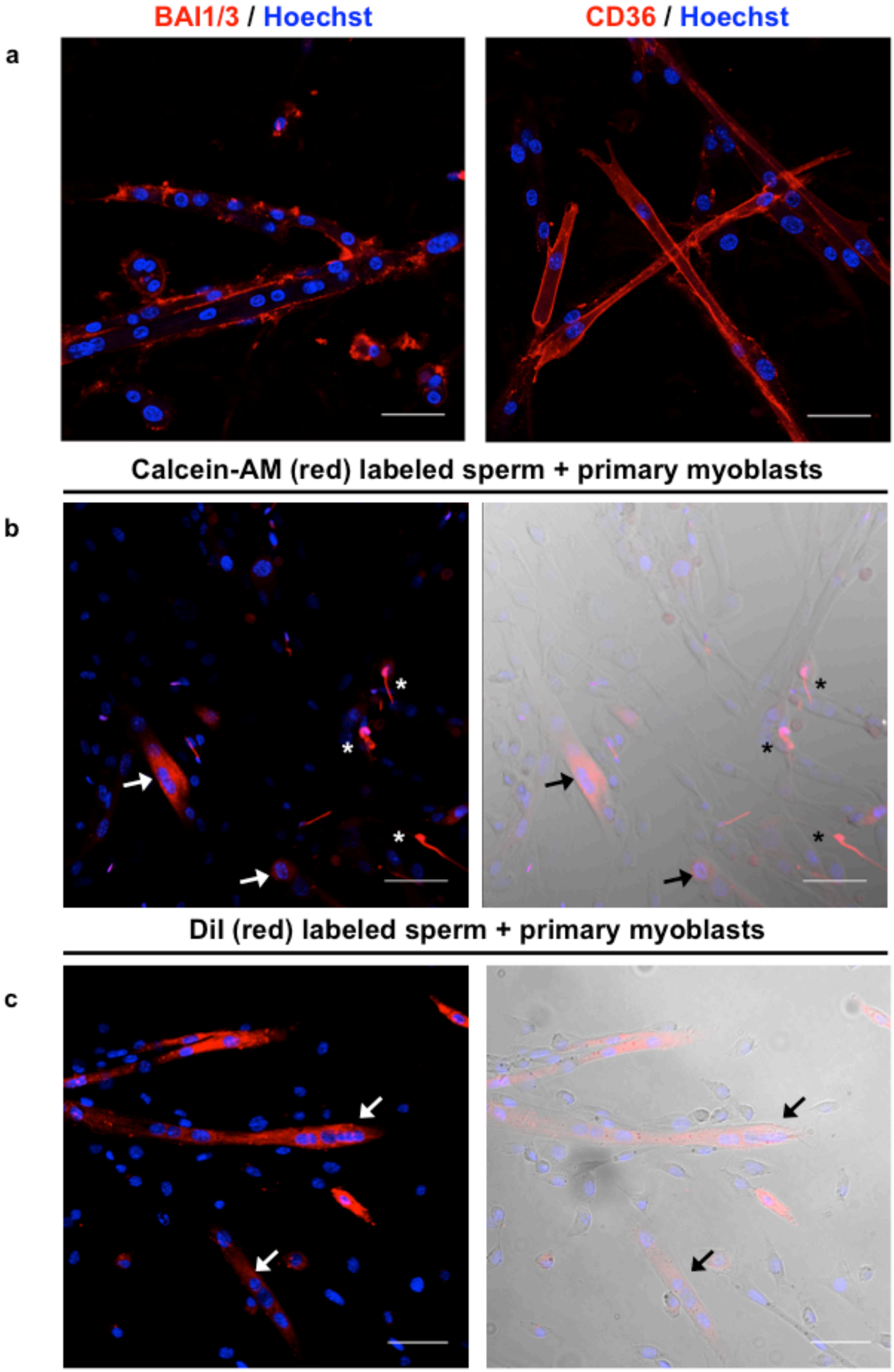

BAI1/3 / Hoechst
CD36 / Hoechst
a
Calcein-AM (red) labeled sperm + primary myoblasts
b
DiI (red) labeled sperm + primary myoblasts
c

Supplementary Figure 6. Primary myoblasts express PtdSer receptors and fuse with sperm. a, Primary myoblasts were incubated with BAI1/3 (left) or CD36 (right) antibodies. Cells were fixed and sequentially stained with biotinylated antibodies, streptavidin-Texas Red, and Hoechst. Scale bar, 50 µm. **b,** Primary myoblasts were co-cultured with Calcein-AM labeled sperm for 4h. Arrows: transfer of the calcein dye from sperm to the myoblasts was visualized. Asterisks: residual sperm that has not fused. Scale bar, 50 µm. **c,** Primary myoblasts were co-cultured with DiI-labeled sperm for 4h. Arrows: transfer of the DiI dye from sperm to the myoblasts. Scale bar, 50 µm.

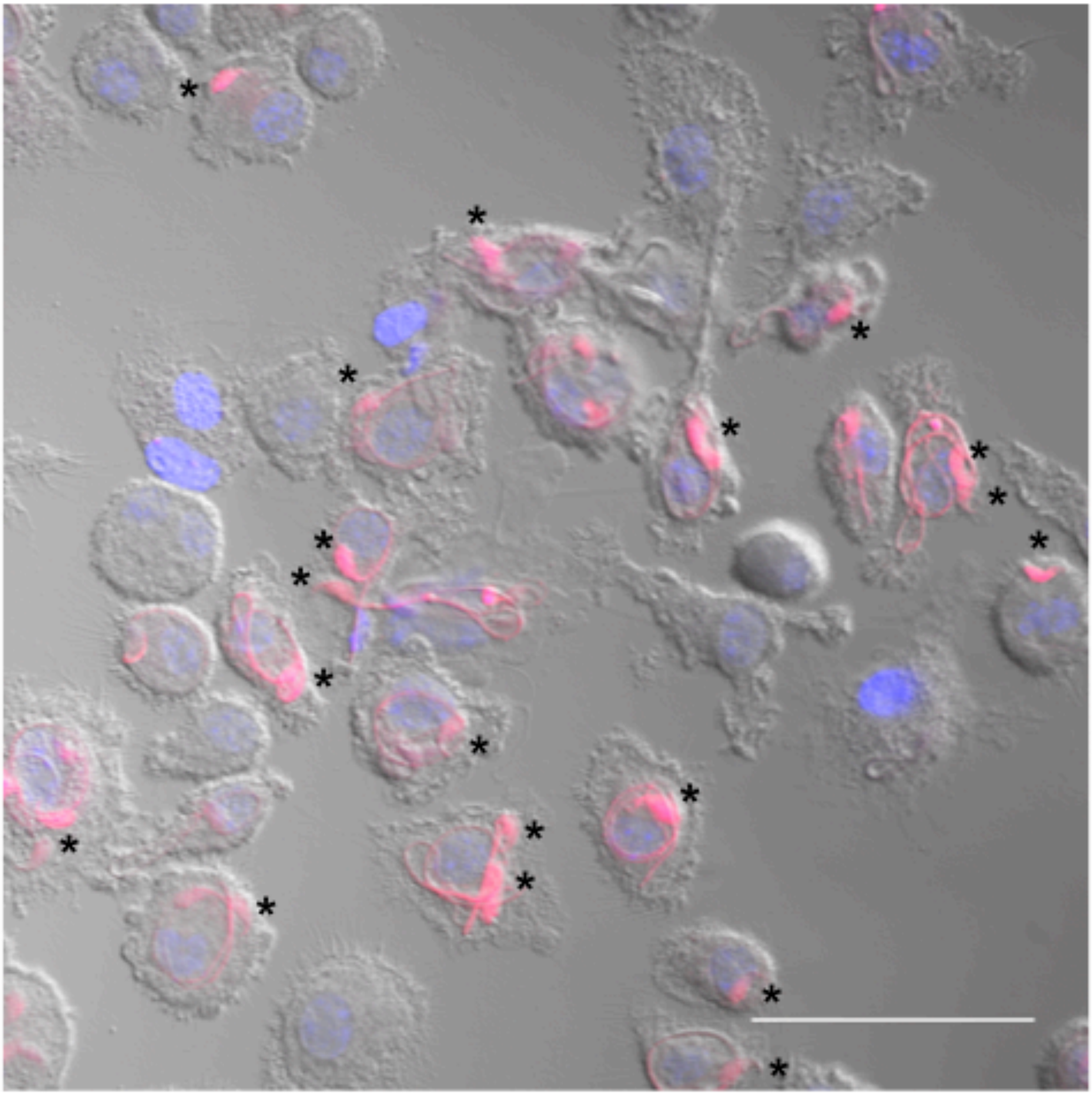

Supplementary Figure 7. Bone marrow derived murine macrophages 'phagocytose' sperm. Calcein-AM labeled sperm was co-incubated with bone marrow derived macrophages. After 4h, multiple sperm were observed within the macrophages. Please note that the dye transfer was not uniform within the cytoplasm as seen with myoblasts, and appears to be within endosomes. Asterisks: sperm heads. Scale bar, 50 μm.

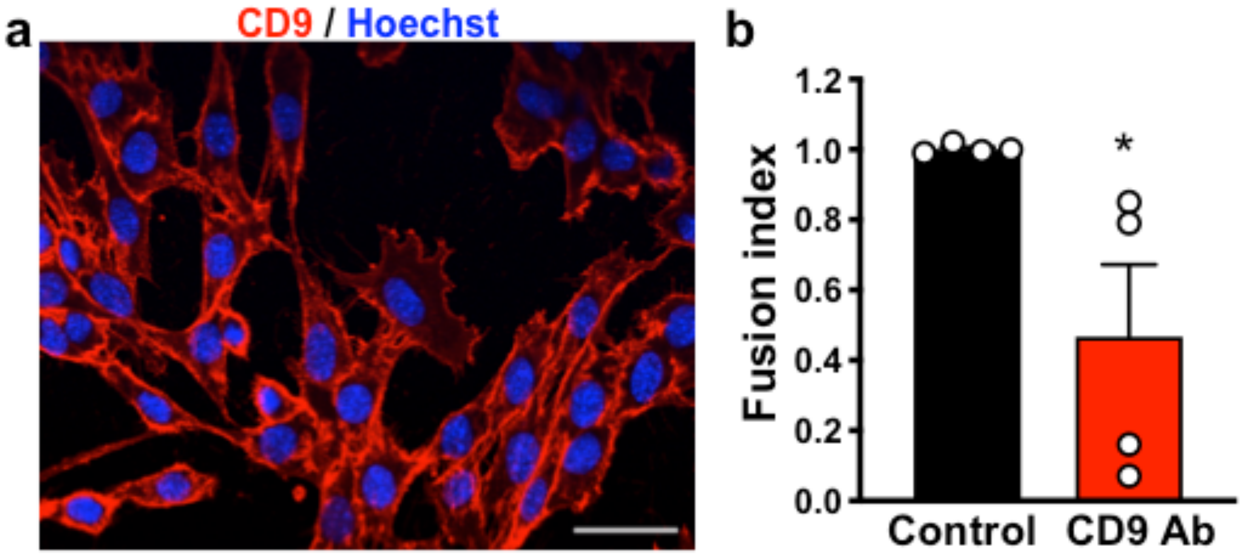

Supplementary Figure 8. CD9 on myoblasts and participates in sperm:myoblast fusion. a, Live C2C12 myoblasts (cultured in growth medium that does not support differentiation into myotubes) were stained with anti-CD9 antibody (clone KMC8), fixed, and stained with a secondary antibody (red) and the nuclei were stained with Hoechst (blue). Bar: 50 µm. **b**, CD9 antibody inhibits sperm:myoblast fusion. Calcein-AM labeled sperm were co-cultured with C2C12 myoblasts previously incubated with a CD9 antibody (clone KMC8). Fusion was evaluated as specified for main Figure 4 in the manuscript. Each dot represents on experiment (n=4 independent experiments). Mean ± s.em.; *p<0.05 (Two tailed unpaired Student's t test). Source Data are provided in the Source Data File.
