# Supplementary material for: Phosphatidylserine on viable sperm and phagocytic machinery in oocytes regulate mammalian fertilization
Source: Nat Commun. 2019 Oct 1;10:4456. doi: 10.1038/s41467-019-12406-z (PMC6773685; doi:10.1038/s41467-019-12406-z)
Supplement: Supplementary file 2 — Description of Additional Supplementary Files [file 41467_2019_12406_MOESM2_ESM.pdf]

Description of Additional Supplementary Files

File name: Supplementary Movie 1
Description: Sperm were isolated from the cauda epididymis, capacitated for 90 minutes in TYH+BSA, and stained with Annexin V conjugated with Alexa Fluor 488 (green). An aliquot of the sperm was placed on 20 □m chamber slides an[illegible]
Movies were recorded and analyzed. Asterisk points to one Annexin V+ spermatozoa (head staining) showing progressive motility.
